# Modernization of Enoxaparin Molecular Weight Determination Using Homogeneous Standards

**DOI:** 10.3390/ph10030066

**Published:** 2017-07-22

**Authors:** Katelyn M. Arnold, Stephen J. Capuzzi, Yongmei Xu, Eugene N. Muratov, Kevin Carrick, Anita Y. Szajek, Alexander Tropsha, Jian Liu

**Affiliations:** 1 Division of Chemical Biology and Medicinal Chemistry, Eshelman School of Pharmacy, University of North Carolina, Chapel Hill, NC 27599, USA; arnoldk2@email.unc.edu (K.M.A.); sc464303@email.unc.edu (S.J.C.); yongmeix@email.unc.edu (Y.X.); murik@email.unc.edu (E.N.M.); alex_tropsha@unc.edu (A.T.); 2 U.S. Pharmacopeia, Rockville, MD 20852, USA; klc@usp.org (K.C.); anita.szajek@nih.gov (A.Y.S.); 3 Center for Scientific Review, National Institutes of Health, Bethesda, MD 20892, USA

**Keywords:** enoxaparin, USP, MW determination, oligosaccharide calibrants, computational modeling, HPLC, compendial test

## Abstract

Enoxaparin is a low-molecular weight heparin used to treat thrombotic disorders. Following the fatal contamination of the heparin supply chain in 2007–2008, the U.S. Pharmacopeia (USP) and U.S. Food and Drug Administration (FDA) have worked extensively to modernize the unfractionated heparin and enoxaparin monographs. As a result, the determination of molecular weight (MW) has been added to the monograph as a measure to strengthen the quality testing and to increase the protection of the global supply of this life-saving drug. The current USP calibrant materials used for enoxaparin MW determination are composed of a mixture of oligosaccharides; however, they are difficult to reproduce as the calibrants have ill-defined structures due to the heterogeneity of the heparin parent material. To address this issue, we describe a promising approach consisting of a predictive computational model built from a library of chemoenzymatically synthesized heparin oligosaccharides for enoxaparin MW determination. Here, we demonstrate that this test can be performed with greater efficiency by coupling synthetic oligosaccharides with the power of computational modeling. Our approach is expected to improve the MW measurement for enoxaparin.

## 1. Introduction

**Figure 1:**
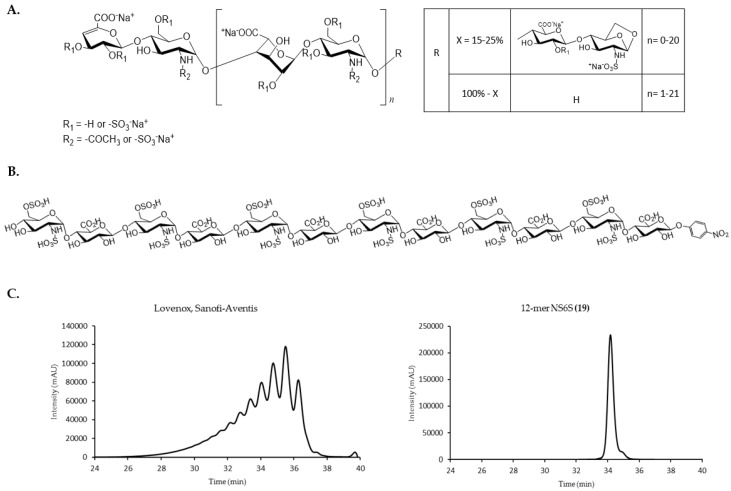
(**A**) Enoxaparin structure. The 1,6-anhydro reducing end is depicted in the box on the right; (**B**) the structure of 12-mer NS6S (**19**); (**C**) representative size exclusion chromatography (SEC) chromatogram of Lovenox and 12-mer NS6S (**19**). Enoxaparin’s heterogeneity and complexity is evident from multiple peaks with poor base-line separation.

Heparin is a mixture of glycosaminoglycan (GAG) chains originating from porcine intestinal mucosa. It is used therapeutically as an anticoagulant for the treatment and prevention of thrombosis [1]. Heparin’s GAG chains are heterogeneous in nature and vary in molecular weight (MW), chain length, degree of sulfation, disaccharide unit composition, and pharmacological effects [2]. The variability in unfractionated heparin’s pharmacokinetic properties and pharmacological effects led to the development of low MW heparin (LMWH), which is a degraded product of heparin using chemical or enzymatic cleavage techniques [2]. LMWHs are now considered the standard of care for the clinical management of venous thromboembolism [1,2]. The most common form of LMWH in the U.S. is enoxaparin, which is produced by β-eliminative cleavage of the benzyl esters of porcine mucosal heparin under alkaline conditions [2]. This cleavage process leads to the generation of unnatural structures in enoxaparin (Figure 1A). The majority of the resulting chains have an unsaturated uronate residue at the non-reducing end, which can be utilized for UV detection at 232 nm, and up to 25% of chains have a 1,6-anhydro structure at the reducing end [3].

While heparin drugs have been used extensively in the clinic since the 1930s, the characterization, regulation, and quality control aspects of these materials remain challenging [4]. Since chain length is one of the factors that affects LMWH biological activity [5], an accurate method to determine MW distribution and average weight MW (*M_w_*) is an essential quality control step. However, due to their high degree of structural complexity and acidity, the determination of the *M_w_* and MW distribution of enoxaparin is not readily amenable to analysis by mass spectrometry [6]. An alternative chromatographic method was developed and added to the US Pharmacopeia (USP) monographs [7,8]. The accuracy of this chromatographic analysis relies ultimately on calibration with standards of high purity [8]. Due to the lack of structurally defined calibrants, the evaluation of *M_w_* and MW distribution represents a controversial aspect of LMWH characterization [5].

Ideal standards for analyzing enoxaparin would be oligosaccharides that encompass enoxaparin’s MW range in order to determine *M_w_* ~4500 Da [9]. In practice, pure oligosaccharide standards of this size are difficult to isolate. Thus, a mixture of calibrants enriched in particular MW ranges have been used. While the disaccharide composition of these calibrants is known, the precise sequences of the intact oligosaccharide chains are unknown, raising a concern about the reproduction of the calibrants. The goal of this study is to develop a new technique that is reliable and sustainable for the determination of *M_w_* and MW distribution by combining synthetic oligosaccharides and computational modeling. To achieve this goal, the following aims were accomplished; (i) the synthesis of a panel of oligosaccharides covering the enoxaparin MW range between 2226 and 5176 Da; (ii) the development of a predictive computational model for MW analysis; (iii) the development of a system suitability protocol; and (iv) the analysis of commercially available Enoxaparin Sodium from different manufacturers.

## 2. Results

### 2.1. Chemoenzymatic Synthesis of Oligosaccharide Panel

A total of 27 oligosaccharides were synthesized by a chemoenzymatic approach, and the MW of each compound was verified by mass spectrometry [10,11]. The compounds are organized into five structural groups (A to E) (see Table 1 and Supplementary Figure S1) based on repeating disaccharide structural units. Group A contains compounds without sulfation; all glucosamine residues are *N*-acetylated. Group B contains compounds with one *N*-sulfo group per disaccharide unit. Group C contains compounds with two sulfo groups per disaccharide unit, with *N*-sulfated glucosamine and 2-*O*-sulfo iduronic acid residues. Group D contains compounds with two sulfo groups per disaccharide unit due to repeating units of *N*, 6-*O*-sulfated glucosamine. Finally, group E contains compounds with three sulfo groups per disaccharide unit due to repeating units of *N*, 6-*O*-sulfated glucosamine and 2-*O*-sulfo iduronic acid.

Additionally, two commercially available heparin oligosaccharides, designated as group F (Iduron, Manchester, UK), were used to address the lower MW range. These oligosaccharides are prepared from partial heparin lyase degradation of heparin, followed by high-resolution gel filtration chromatography purification. The general structure is ∆HexA2S-GlcNS6S-(IdoA2S-GlcNS6S)_n_, with *n* = 1 for dp4 and *n* = 2 for dp6. Although not homogeneous, the majority (~75%) of structures contain three sulfo groups per disaccharide unit, consisting of *N*, 6-*O*-sulfated glucosamine and 2-*O*-sulfo iduronic acid.

The USP Enoxaparin Sodium MW calibrant A and B materials (USP, Rockville, MD, USA), designated as group G, were included in this study (Table 1 and Figure S2). The reported MW values in the calibrants for each chromatographic peak are 11000, 7750, 5200, 3350, 2250, 1800, and 1400 Da.

Compared to enoxaparin, synthetic oligosaccharides have defined structures, as demonstrated by **19** in Figure 1B. All synthetic oligosaccharides (**1** to **27**) migrated as a single, narrow main peak on size exclusion chromatography (SEC), supporting their homogeneous nature (Figure S1). These oligosaccharides can be reliably reproduced based on the established synthetic schemes with minimal impurities.

### 2.2. Size Exclusion Chromatography Profiles of Synthetic Oligosaccharides

#### 2.2.1. SEC Characterization of Synthetic Oligosaccharides

**Figure 2:**
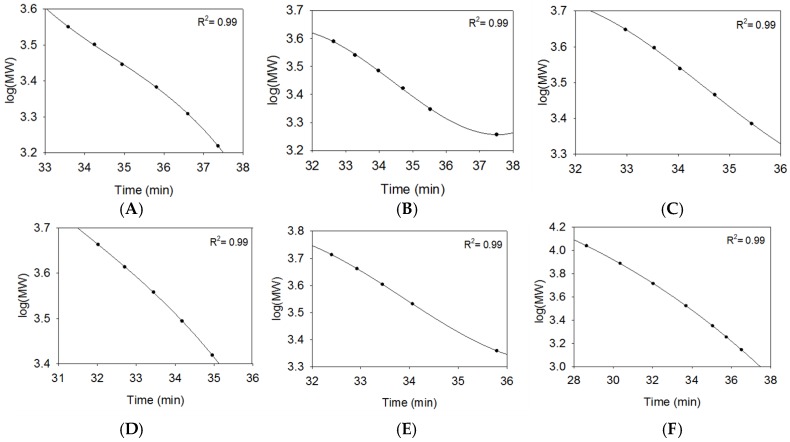
Calibration curves for determining *M_w_* generated from different structural groups: (**A**) NAc series (group A); (**B**) NS series (group B); (**C**) NS2S series (group C); (**D**) NS6S series (group D); (**E**) NS6S2S series (group E); (**F**) USP Calibrant A and B (group G). The nonlinear regression R^2^ value is listed in upper right corner of each panel.

The USP compendial method uses size exclusion chromatography (SEC) to relate retention time (RT) to MW. The synthetic oligosaccharides were evaluated for use as reference standards by obtaining a RT via SEC (Figure S1). The analyses were conducted in five groups, group A to E, based on the structural features of these samples. Following the compendial method, a calibration curve for each structural group was generated by plotting log(MW) versus RT and used for nonlinear regression analysis (Figure 2A–E). USP calibrants, as well as the synthetic oligosaccharides in each structural group, exhibited tight correlation between the MW and RT (Figure 2).

#### 2.2.2. SEC RT Depends on Both Oligosaccharide Shape and Size (MW)

We next compared the calibration curves from each structural group to that of the USP calibrants by superimposing the analyses (Figure S3). Two main observations are apparent; (i) the structure of the oligosaccharides affects the RT and (ii) smaller synthetic oligosaccharides do not behave similiarly to enoxaparin-like materials.

**Figure 3:**
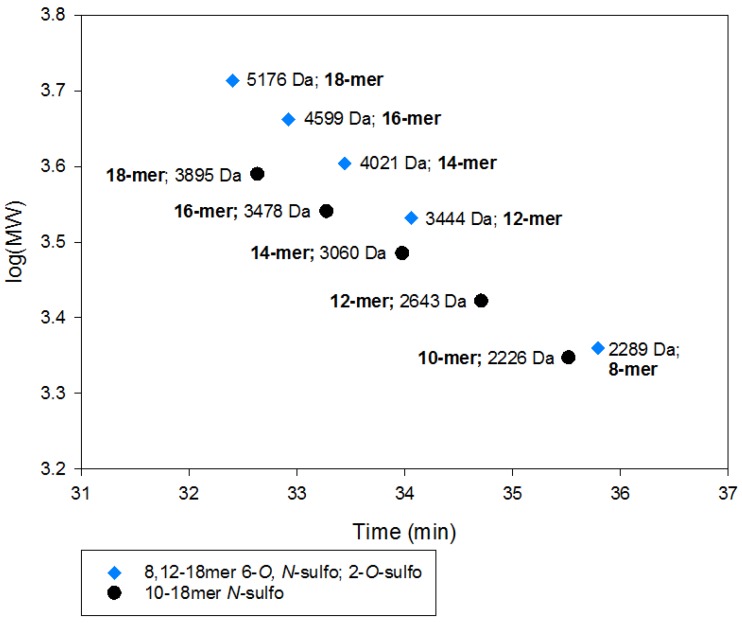
Comparison of two structural series and SEC behavior. Blue diamonds are group E (NS6S2S series). Black circles are group B (NS series).

Oligosaccharides containing sulfo groups (Figure S3B–E) migrated more closely to the USP calibration curve than group A, the non-sulfated NAc series (Figure S3A), even though each group is comprised of similar MWs. This result suggests that the structures of oligosaccharides impact the RT. This observation raises the possibility that saccharide sequences carrying different sulfo groups or IdoA residues may change the overall oligosaccharide shape and display different SEC migration properties. The assertion is further demonstrated in Figure 3 by comparing group E oligosaccharides (NS6S2S series) with group B oligosaccharides (NS series). Here, the effect on RT becomes noticeable for those oligosaccharides that have a MW greater than 3000 Da. Larger oligosaccharides in group E display similar RT to smaller oliogsaccharides in group B, despite a clear difference in MW. One possible expanation is that group E oligosaccharides contain conformationally flexible IdoA residues, underlining the influence of the molecular shape of oligosaccharides on RT. It is noted that both 1,6-anhydro glucosamine and 1,6-anhydro mannosamine are present at the reducing end of some oligosaccharide chains of enoxaparin [7]. However, the disaccharide building blocks containing 1,6-anhydro residues only represent <2% of the disaccharide building blocks in enoxaparin. At the present time, we were unable to evaluate the impact of 1,6-anhydro residues on the elution behavior during the analysis.

**Figure 4:**
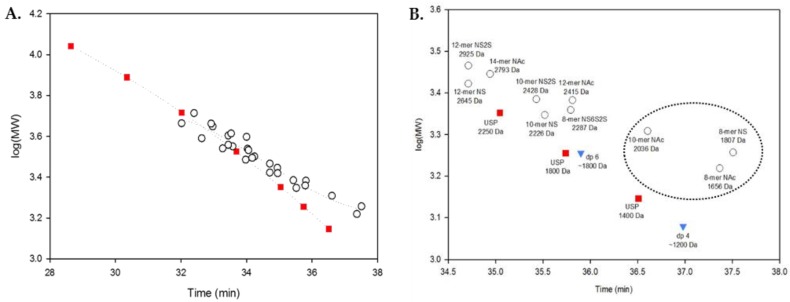
(**A**) USP calibrants (red squares) and all synthetic oligosaccharides (black open circles); (**B**) Correlation of the molecular weight (MW) and retention time (RT) of compounds below 3000 Da annotated. Compounds enclosed in the circle, 8-mer NAc (**1**), 10-mer NAc (**2**), and 8-mer NS (**7**), were the outliers and were excluded from further analysis.

Another observation is that short oligosaccharides do not have similar RTs to enoxaparin-like materials such as the USP calibrants. We compared all 27 oligosaccharides with to the USP calibrants (Figure 4A) and found that the RT values of the smaller synthetic oligosaccharides deviate the greatest from the USP calibrants. To address this lower MW range deviation, two commerically available heparin oligosaccharides, dp6 (6-mer) and dp4 (4-mer), were included (Figure 4B, blue triangles). dp6 and dp4 both closely align with the USP calibrants. For this reason, three synthetic oligosaccharides, 8-mer NAc (**1**), 10-mer NAc (**2**), and 8-mer NS (**7**), were excluded from further analysis, as described below.

### 2.3. Using Calibration Curves to Determine M_w_ of USP Enoxaparin RS

Following the USP compendial method [7], the equation from the nonlinear regression analysis was used to determine the *M_w_* of USP Enoxaparin RS. Using the generated USP calibration curve, the measured *M_w_* is very close to the reported *M_w_* for USP Enoxaparin RS (Table 2, Row 1), suggesting that the procedures were appropriately carried out. However, all of the determined *M_w_* values are outside the USP acceptable range of ±3.4% (Table 2, Rows 2–7). Using all 24 synthesized oligosaccharides, one would expect that the *M_w_* would be accurately determined since the major structural components are accounted for; however, the measured *M_w_* was 3250 Da, which was still 25.6% lower than the anticipated value of 4370 Da.

The unexpected difference in measured *M_w_* values is likely due to the selection of the calibrants/standards for the analysis. The current USP method was developed using calibrants with heterogeneous structures, while our method uses homogeneous standards. To amend the difference, the relative molar contributions of the disaccharide repeating units of enoxaparin are taken into account. As shown in Table 3, enoxaparin is comprised of a mixture of compounds in which ~89% show similarity to one of our five oligosaccharide structural groups [12]. The remaining 11% of components in enoxaparin includes several structures, each contributing less than 2% to the overall mixture composition and for which we do not have complementary synthetic oligosaccharides. When using all the synthetic oligosaccharides via the nonlinear regression method to determine *M_w_*, each oligosaccharide contributed equally to the overall *M_w_*, even if the structure only accounted for a very small molar percentage. Therefore, we turned to computational tools to allow us to use these homogeneous compounds appropriately as calibrants that account for enoxaparin’s heterogeneity during analysis.

### 2.4. Method Modernization: SVM Modeling Allows for Synthetic Oligosaccharides to be Used in the Appropriate Proportions to Reflect the Various Quantities of Components in Enoxaparin

We used a Support Vector Machine (SVM) [13] technique for the modeling, in place of the nonlinear regression analysis recommended by the USP. The nonlinear regression assumes that the relationship of MW and RT fits a cubic function. On the other hand, the SVM model learns statistically meaningful relationships between MW and RT, approximates a multidimensional regression function, and optimizes this relationship for predictions. Moreover, unlike the standard nonlinear regression analysis, which treats all data points equally, the SVM model can assign greater statistical importance to specific data points. In our case, several oligosaccharides have nearly identical RTs, yet their MWs are different. If an enoxaparin sample displays a peak at this RT, the MW of the peak is calculated based on the relative abundance of the distinct oligosaccharides.

**Figure 5:**
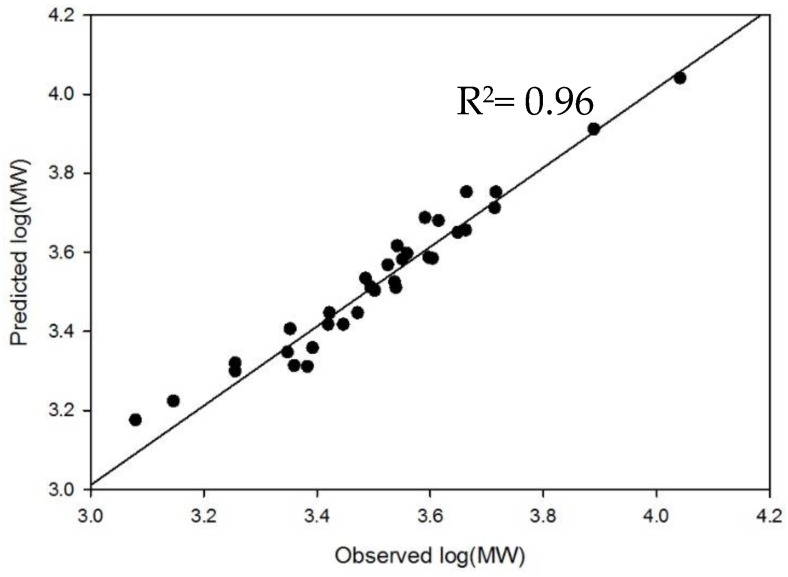
Predicted log(MW) versus observed log(MW) to demonstrate the strength of the model’s prediction. For predicted log(MW), only external set MW predictions are used. Observed MW is determined by MS for the pure oligosaccharides. For dp4, dp6, and USP calibrants, the provided MW values were used as the observed MW. The model’s predictions highly correlate to the expected results (R^2^ = 0.96).

A total of 33 compounds were included in the training set, including 24 synthetic compounds, seven current USP calibrant data points, and the two purchased oligosaccharides (Table S1). The compounds were represented based on their repeating disaccharide unit to more closely reflect enoxaparin in a process known in statistical modeling as ‘weighting’. Group E and F oligosaccharides (NS6S2S, dp4 and dp6) are weighted by a factor of 10, followed by group D oligosaccharides (NS6S series), which were weighted by a factor of four, and group C oligosaccharides (NS2S series), which were weighted by a factor of two. While group A, B, and G (NAc, NS and USP calibrants) remain unweighted in the data set, their inclusion is important in order to adequately capture the low and high ends of the MW range. To visualize how well the model predicts MW, the distribution of the predicted versus observed MW values of the external set compounds is shown in Figure 5. As part of the validation of the model, each compound in the training set is systematically left out of model building and placed into an external set. The resulting model then predicts the compounds in the external set.

The first level of external model validation was performed by determining the *M_w_* of USP Enoxaparin RS. Unlike our nonlinear regression analysis, the SVM model is able to successfully predict *M_w_* within the acceptable range, as seen in Table 4.

The largest oligosaccharide that can currently be synthesized by the chemoenzymatic synthesis approach is 18 residues long (~5000 Da), yet enoxaparin’s MW distribution expands beyond 5000 Da. This is not an issue, though, when the SVM model is used for analysis because, in addition, to achieving an acceptable *M_w_* value, the predicted MWs for the largest components in USP Enoxaparin RS are comparable with the results from the USP calibrants up to 11,000 Da (Table S2). The determination of MWs above 11,000 Da requires extrapolation; therefore comparison between the methods at this point must be done with care.

These results demonstrate that the utility of the synthetic oligosaccharides can only be fully realized when coupled with an appropriate computational model. In this way, we are able to reflect the nature of the enoxaparin mixture by weighting oligosaccharides based on their relative molar contribution to the composition of the enoxaparin mixture.

### 2.5. System Suitability: Applying the SVM Model in a Compendial Technique

The use of the SVM model eliminates the need to run all oligosaccharide standards for analysis, thereby shortening the analysis. Currently the SVM model is a stand-alone tool that only requires RT and peak areas as an input. Ultimately, we envision a software interface that houses this SVM model to facilitate a streamlined analysis process. Any future software development that incorporates the validated SVM model will contain a system suitability assessment before access to the model is granted.

**Figure 6:**
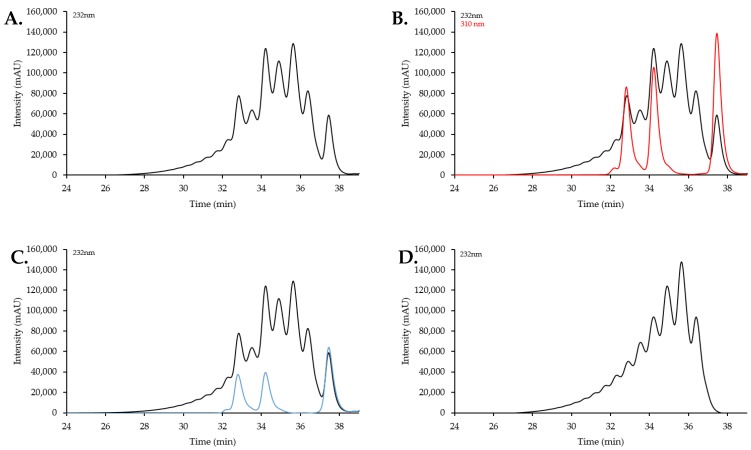
(**A**) 232 nm trace of enoxaparin mixed with three oligosaccharides. Although primary detection of the synthetic oligosaccharides is at 310 nm, there is some residual absorbance also at 232 nm, as evidenced by the abnormal profile compared to Figure 1C; (**B**) Overlay of 310 nm and 232 nm trace from a single run of the enoxaparin/oligosaccharide mixture. 310 nm peak RTs (red trace) were used to determine the suitability of the HPLC system by comparing them with the predetermined 310 nm chromatogram from oligosaccharides alone (Figure S4); (**C**) Overlay of predetermined 232 nm trace (blue trace); (**D**) The resulting 232 nm trace when the oligosaccharide contribution is removed. The peak RTs and area values are input into the model for analysis.

A system suitability test is required in the USP compendial method to ensure that the selected analytical technique offers the adequate resolution to analyze enoxaparin. Using the oligosaccharides coupled with the SVM model, we can accomplish the system suitability test and analysis in one HPLC process by using two detection wavelengths; 232 nm for enoxaparin and 310 nm for the oligosaccharides (Figure 6). To this end, enoxaparin was mixed with three synthetic oligosaccharides, 8-mer NS (**7**), 12-mer NS6S (**19**), and 16–mer NS6S (**21**), for SEC analysis to obtain a chromatogram shown in Figure 6A. A system suitability check was performed by confirming the RTs of the internal oligosaccharide standards at 310 nm (Figure 6B). The 310 nm RTs fell within the acceptable range of ±0.1 min, predetermined by running the oligosaccharides alone. As shown in Figure S4, the overlapped RT values indicated that the system is suitable for use with the SVM model. Next, the peak areas contributed by the oligosaccharides at 232 nm (Figure 6C, blue trace) were subtracted from the chromatogram. The resulting 232 nm chromatogram is used by the SVM model for the determination of the *M_w_* and the MW distribution (Figure 6D).

### 2.6. Analysis of Commercially Available Enoxaparin Sodium Using Oligosaccharide SVM Model

Using the SVM model and system suitability procedure, three commercially available Enoxaparin Sodium samples, both brand and generic versions, were analyzed. The *M_w_* and MW distribution results are shown in Table 5. The results demonstrate that our approach allows for consistent and adequate testing of *M_w_*. We acknowledge that our UV detection method differs from the refractive index (RI) detection method listed in the monograph. We chose UV detection because the oligosaccharides have a tag that absorbs at 310 nm with very high sensitivity. Although RI detection can be used in principle, large quantities of the oligosaccharide standards will be needed due to the RI’s low detection sensitivity. This would unnecessarily increase the cost of analysis. The difference in detection methods may account for the consistently high percentage between 2000–8000 Da. The harmonization of RI and UV detection can be achieved in subsequent studies after analyzing a large set of enoxaparin samples.

## 3. Discussion

LMWHs are heterogeneous mixtures of oligosaccharides, the MW distribution of which can be described by *M_w_* and by identifying the percentage of material that falls within specificied MW ranges [6]. The determination of these parameters is an important step in enoxaparin quality control and requires reliable, pure calibrants. Since the RT from SEC is the result of both a molecule’s size/weight and shape, the calibrants must closely reflect the structure of enoxaparin [14]. However only a portion of its natural oligosaccharides have been isolated and characterized [15], leaving the rest of the mixture largely unknown. There are several reports on the disaccharide composition of heparin and enoxaparin, which sheds light on proportions of repeating structural units found in these complex mixtures [16,17,18]. With this in mind, we report the generation of enoxaparin-like oligosaccharides via chemoenzymatic synthesis. The panel of compounds is comprehensive both in the scope of MW and structural class coverage, which is important when considering the influence of both MW and shape on RT.

After SEC characterization, it became evident that the synthetic oligosaccharides cannot be simply substituted for the existing USP calibrants without changing the procedures for data analysis. Surprisingly, although each structural series fits its own regression lines closely, no individual structural group was able to correctly determine the *M_w_* for USP Enoxaparin RS (Table 2, Rows 2–7). For example, using the group A calibration curve, the measured *M_w_* was 22300 Da, 410% above the label value, whereas using the group B calibration curve, the measured *M_w_* was 3100 Da, 29.1% below the anticipated value. The deviation of the calibration curves using synthetic oligosaccharides and USP calibrants (Figure S3) suggests that the homogeneous oligosaccharides did not fully represent the migration properties of the USP calibrants that contain mainly heterogeneously sulfated oligosaccharides. The current compendial method is designed for specific use with the intended USP calibrants, and replacement with synthetic oligosaccharides does not give the correct *M_w_* for USP Enoxaparin RS. Therefore, we needed to amend the analysis method to accommodate our homogeneous compounds.

Using structurally defined oligosaccharides as MW standards for SEC analysis is undoubtedly a superior approach compared to a mixture of oligosaccharides since these homogeneous standards can be accurately re-synthesized. However, implementation of the pure oligosaccharides requires careful consideration. Our study represents an important first step towards using these materials in practice. The key finding from our study is that a combination of a set of synthetic oligosaccharides and a computational model for data processing is essential for predicting the *M_w_* of enoxaparin. SVM modeling techniques allowed us to use the panel of synthetic oligosaccharides in appropriate proportions based on their relative abundance in enoxaparin, with groups E and F weighted the greatest, followed by groups C and D. Groups A, B, and G are included but not weighted in the data set in order to cover a large MW range. Without coverage of the extremes of the range, the extrapolative ability of the SVM model would be hampered. Therefore, these compounds are needed to capture a broad MW range so that this model can be extended to other heterogeneous heparin materials (i.e., bovine sourced preparations) in the future. To further demonstrate the robustness of this method, more enoxaparin lots from different manufacturers will be analyzed in future studies.

Our approach is also more efficient both in terms of time and cost compared to the current method (Table 6). The overall time from injection onto the HPLC to the final result is dramatically reduced since only one analysis (enoxaparin sample mixed with oligosaccharides) needs to be run. In comparison, the current method requires running several individual materials (MW Calibrant A and B, RS, and an enoxaparin sample). With further software development, the process can become automated. For example, the software will house the SVM model in such a way that the *M_w_* and the distribution are automatically reported after input data passes the system suitability check, thereby eliminating operators’ subjectivity during the analysis. We envisage that our approach will improve the quality control for enoxaparin production. Given that numerous structurally heterogeneous drugs exist on the marketplace, our strategy, using homogenous standards to calibrate a mixture of heparin, can be potentially expanded to analyze these complex drugs.

## 4. Materials and Methods

### 4.1. Chemoenzymatic Synthesis

The panel of oligosaccharides, ranging in length from octasaccharide (8-mer) to octadecasaccharide (18-mer), was synthesized using an established chemoenzymatic approach [10]. Briefly, each compound was synthesized from a commercially available glucuronide-*para*-nitrophenyl monosaccharide starting material, followed by a series of elongation steps and subsequent epimerization and sulfation modifications catalyzed by recombinant enzymes. Structural characterization was confirmed by nuclear magnetic resonance (NMR) and electrospray ionization mass spectrometry (ESI-MS), as previously described [10].

### 4.2. MW Distribution and M_w_

The USP compendial method for the determination of enoxaparin *M_w_* and MW distribution utilizes SEC. The reported method is adapted from the compendial method stated in the Enoxaparin Sodium monograph [7]. The apparatus was composed of a Shimadzu LC-20AB pump, a Shimadzu SIL-20A HT auto sampler, a Shimadzu CBM-20A controller, and a Shimadzu SPD-M20A diode array detector (Shimadzu, Kyoto, Japan). The following chromatography columns were connected in series; TSKgel guard column SWxl (6 mm I.D. × 4 cm), TSKgel G2000 SWxl (7.8 mm I.D × 30 cm), and TSKgel G3000 SWxl (7.8 mm I.D. × 30 cm). The mobile phase consisted of 0.1 M ammonium acetate and 0.5 M NaCl, filtered through 0.22 micron membrane, at a flow rate of 0.6 mL/min. Samples were filtered through a 0.22 micron membrane and injected in 20 uL volumes.

USP Enoxaparin MW Calibrant A and B (USP, Rockville, MD, USA) were dissolved in mobile phase and run in duplicate to obtain peak RTs. The provided peak MWs were used to plot log(MW) versus RT. USP Enoxaparin RS (USP, Rockville, MD, USA) was dissolved in mobile phase and run in duplicate. The nonlinear regression analysis of log(MW) versus RT (MS Excel, Sigmaplot, Seattle, WA USA) was used to determine the weight-average MW (Equation (1)), *M_w_*, of the USP Enoxaparin RS, which has a label *M_w_* value of 4370+/−150 Da, in order to demonstrate the system’s suitability.

(1) $$M_{w}=\frac{\sum_{i}N_{i}M_{i}^{2}}{N_{i}M_{i}},$$

where *N_i_* is the number of molecules at MW *M_i_*. Although refractive index (RI) detection is listed in the USP monograph, using UV signal intensity from the Calibrant A and B materials with Equation (1) resulted in a determination of the *M_w_* of USP Enoxaparin RS within the acceptable range. UV detection is preferred due to the ability to discriminate the oligosaccharides from enoxaparin in a single sample based on their respective **λ**_max_ values.

The *M_w_* and MW distribution of enoxaparin was determined by dissolving in mobile phase and running in duplicate. The peak areas and total area under the chromatogram, excluding salt and solvent peaks, are used by the model to determine the MW distribution, which is defined by the percentage of enoxaparin with MW less than 2000 Da, *M*_2000_, the percentage between 2000 to 8000 Da, *M*_2000–8000_, and the percentage greater than 8000 Da, *M*_8000_.

The total panel of synthesized oligosaccharides was run individually to obtain a RT for each. Collectively, the MWs and RTs were used to subsequently determine the *M_w_* and MW distribution of USP Enoxaparin RS and the enoxaparin samples.

Commercially available heparin oligosaccharides (Iduron, Manchester, UK) with approximate MWs of 1800 Da and 1200 Da were used to address the MW range below 2000 Da. These RTs were included with the oligosaccharide panel in the nonlinear regression analysis and computational model.

For system suitability tests, 8-mer NS, 12-mer NS6S, and 16-mer NS6S were combined and vortexed to mix. 30 μg of this solution (10 μg of each) was injected in duplicate in order to determine the absorbance at 310 and 232 nm. Then an enoxaparin sample (1 mg) was combined with the oligosaccharide solution and vortexed to mix. From the resulting solution, a single injection containing 0.2 mg of enoxaparin and 10ug of each oligosaccharide was injected in duplicate, and chromatograms were recorded at 310 and 232 nm.

The monograph specifications were used as criteria to evaluate the effectiveness of oligosaccharide nonlinear regression and the SVM model:

- The determination of USP Enoxaparin RS within 150 Da of labeled *M_w_* value.
- The determination of an enoxaparin test sample with *M_w_* ~4500 Da, the range being between 3800 and 5000 Da.
- The determination of an enoxaparin test sample MW distribution:
  ○ ~16.0% below 2000 Da, the range being between 12.0 and 20.0%;
  ○ ~74.0% between 2000 and 8000 Da, the range being between 68.0 and 82.0%;
  ○ Not more than 18.0% higher than 8,000 Da.

### 4.3. Development and External Validation of SVM Model

The training set of the model contains 33 unique samples;–6, 8–29, and the current USP calibrants (seven data points). Based on the relative molar percent contribution to the overall enoxaparin mixture the NS6S2S, NS6S, and NS2S oligosaccharides, as well as the purchased oligosaccharides were weighted. The NS and NAc oligosaccharides and the current USP calibrants were not weighted.

Each of these 33 samples has an experimentally derived RT and an associated MW. Thus, the MW is the endpoint to be predicted by the model, and the RT of each sample is the input descriptor used to establish the statistical relationship. The machine learning algorithm utilized in the model is a Radial-Basis Function Support Vector Machine (RBF-SVM). During the generation and validation of the model, external leave-one-out cross validation (LOO-CV) was performed and the overall q^2^ (LOO-CV regression) was calculated and deemed statistically valid [19,20]. Y-randomization [21] was performed to ensure that a statistically meaningful relationship between MW and RT exists.

An additional round of external validation [21] was performed to ensure the predictivity of the model using samples obtained from commercially available Enoxaparin Sodium; Lovenox™, Sanofi-Aventis, Bridgewater, NJ; Enoxaparin Sodium Injection, Sandoz, Princeton, NJ; and Enoxaparin Sodium Injection, Amphastar Pharmaceuticals, Rancho Cucamonga, CA. Each sample was from the same lot and was run five times. Data are presented as an average ± standard deviation of *M_w_* and its distribution. These samples were not used as training set samples during model building, thereby constituting purely external data points. The RTs of these samples were used as model inputs, and a MW prediction was made.

## 5. Conclusions

The determination of the MW distribution and the *M_w_* of enoxaparin is an essential yet challenging quality control step that relies on calibrants to ensure the quality and consistency of the product. Chemoenzymatic synthesis allows for a reproducible production of pure oligosaccharides that can be used as standards for a MW analysis of enoxaparin. We employed 27 oligosaccharides that vary in MW and structure in order to match the range of possible compounds present in enoxaparin. Following the nonlinear regression analysis, we were unable to achieve accurate results. We addressed this issue by utilizing an SVM computational technique. Using the oligosaccharides and the current USP calibrants as a training set, we succeeded in developing a robust SVM model for efficient analysis by appropriately weighting oligosaccharides based on their relative abundance in enoxaparin. In addition to standard external validation, we have proven the predictive power of the model by analyzing both brand and generic versions of Enoxaparin Sodium. For the most effective use in practice, we recommend first performing a system suitability check, in which three designated oligosaccharides are run to ensure that the HPLC conditions are adequate, before the subsequent use of the developed SVM model.

We recognize that using a different HPLC detection method than what is stated in the monograph is a limitation of this work. The current calibrants, RS materials, and acceptability criteria have been established using RI detection. Therefore, it is not surprising that our results (Table 5) are not in exact agreement with the monograph criteria. In this study, we demonstrate the proof-of-concept that homogenous oligosaccharides can be used for MW determination. To further advance this promising approach, future studies will focus on (i) harmonization between RI and UV detection to ensure accuracy when comparing the results to the established acceptability criteria; (ii) the determination of the limits of SVM model in terms of HPLC variability; (iii) the development of a user-interface software to house the SVM model; and (iv) the extension of the method to compare to other countries’ pharmacopeias, namely the European Pharmacopeia. As a result, our work provides a sustainable and efficient method for performing this compendial test for enoxaparin and represents a promising approach that could improve quality testing in the future.

## Figures and Tables

**Table 1:** Panel of oligosaccharides and US Pharmacopeia (USP) Enoxaparin MW calibrant A and B materials.

Group	Compound Name	Repeating Unit Structure ^1^	MW (Da)
A.	8-mer NAc (**1**)	GlcNAc-(GlcA-GlcNAc)_3_-GlcA-pNP	1656
	10-mer NAc (**2**)	GlcNAc-(GlcA-GlcNAc)_4_-GlcA-pNP	2036
	12-mer NAc (**3**)	GlcNAc-(GlcA-GlcNAc)_5_-GlcA-pNP	2415
	14-mer NAc (**4**)	GlcNAc-(GlcA-GlcNAc)_6_-GlcA-pNP	2794
	16-mer NAc (**5**)	GlcNAc-(GlcA-GlcNAc)_7_-GlcA-pNP	3174
	18-mer NAc (**6**)	GlcNAc-(GlcA-GlcNAc)_8_-GlcA-pNP	3553
B.	8-mer NS (**7**)	GlcNS-(GlcA-GlcNS)_3_-GlcA-pNP	1808
	10-mer NS (**8**)	GlcNS-(GlcA-GlcNS)_4_-GlcA-pNP	2226
	12-mer NS (**9**)	GlcNS-(GlcA-GlcNS)_5_-GlcA-pNP	2643
	14-mer NS (**10**)	GlcNS-(GlcA-GlcNS)_6_-GlcA-pNP	3060
	16-mer NS (**11**)	GlcNS-(GlcA-GlcNS)_7_-GlcA-pNP	3478
	18-mer NS (**12**)	GlcNS-(GlcA-GlcNS)_8_-GlcA-pNP	3895
C.	10-mer NS2S (**13**)	GlcNS-GlcA-(GlcNS-IdoA2S)_3_-GlcNS-GlcA-pNP	2466
	12-mer NS2S (**14**)	GlcNS-GlcA-(GlcNS-IdoA2S)_4_-GlcNS-GlcA-pNP	2963
	14-mer NS2S (**15**)	GlcNS-GlcA-(GlcNS-IdoA2S)_5_-GlcNS-GlcA-pNP	3461
	16-mer NS2S (**16**)	GlcNS-GlcA-(GlcNS-IdoA2S)_6_-GlcNS-GlcA-pNP	3958
	18-mer NS2S (**17**)	GlcNS-GlcA-(GlcNS-IdoA2S)_7_-GlcNS-GlcA-pNP	4456
D.	10-mer NS6S (**18**)	GlcNS6S-(GlcA-GlcNS6S)_4_-GlcA-pNP	2626
	12-mer NS6S (**19**)	GlcNS6S-(GlcA-GlcNS6S)_5_-GlcA-pNP	3124
	14-mer NS6S (**20**)	GlcNS6S-(GlcA-GlcNS6S)_6_-GlcA-pNP	3621
	16-mer NS6S (**21**)	GlcNS6S-(GlcA-GlcNS6S)_7_-GlcA-pNP	4118
	18-mer NS6S (**22**)	GlcNS6S-(GlcA-GlcNS6S)_8_-GlcA-pNP	4616
E.	8-mer NS6S2S (**23**)	GlcNS6S-GlcA-(GlcNS6S-IdoA2S)_2_-GlcNS6S-GlcA-pNP	2289
	12-mer NS6S2S (**24**)	GlcNS6S-GlcA-(GlcNS6S-IdoA2S)_4_-GlcNS6S-GlcA-pNP	3444
	14-mer NS6S2S (**25**)	GlcNS6S-GlcA-(GlcNS6S-IdoA2S)_5_-GlcNS6S-GlcA-pNP	4021
	16-mer NS6S2S (**26**)	GlcNS6S-GlcA-(GlcNS6S-IdoA2S)_6_-GlcNS6S-GlcA-pNP	4599
	18-mer NS6S2S (**27**)	GlcNS6S-GlcA-(GlcNS6S-IdoA2S)_7_-GlcNS6S-GlcA-pNP	5176
F.	dp4 (**28**)	∆HexA,2S-GlcNS6S-IdoA2S-GlcNS6S	~1200
	dp6 (**29**)	∆HexA,2S-GlcNS6S-(IdoA2S-GlcNS6S)_2_	~1800
G.	USP A.1		11000
	USP A.2		5200
	USP A.3		2250
	USP A.4		1400
	USP B.1		7750
	USP B.2		3350
	USP B.3		1800

^1^ Abbreviations: GlcA (glucuronic acid); GlcN (glucosamine); GlcNAc (*N*-acetylated glucosamine); GlcNS (*N*-sulfo glucosamine); IdoA2S (2-*O*-sulfo iduronic acid); GlcNS6S (6-*O*, *N*-disulfo glucosamine); HexA (hexauronic acid); -pNP (*para*-nitrophenyl).

**Table 2:** Determination of USP Enoxaparin RS *M_w_*.

Series	USP Enoxaparin RS *M_w_* (4370 ± 150 Da)	Deviation from Acceptability Range (100 ± 3.4%)
USP Calibrants	4300	−1.6%
NAc (group A)	22,300	+410.3%
NS (group B)	3100	−29.1%
NS2S (group C)	3500	−19.9%
NS6S (group D)	3350	−23.3 %
NS6S2S (group E)	3700	−15.3%
All oligosaccharides	3250	−25.6%

**Table 3:** Major structural components of Enoxaparin.

Disaccharide Structure	Molar %
∆UA2S-GlcNS6S	~70%
∆UA-GlcNS6S	~10%
∆UA2S-GlcNS	~6%
∆UA-GlcNS	~2%
∆UA-GlcNAc	~1%

**Table 4:** Validation of oligosaccharide Support Vector Machine (SVM) model.

	USP Calibrants	Oligosaccharide SVM Model
USP Enoxaparin RS (*M_w_* = 4370 ± 150 Da)	4300 Da	4450 Da

**Table 5:** Analysis of commercially available Enoxaparin Sodium using SVM model.

Sample	*M_w_*	Distribution
Lovenox	*M_w_* = 4650 ± 35 Da	M_2000_: 14.0 ± 0.5%
		M_2000–8000_: 82.0 ± 0.5%
		M_8000_: 4.0 ± 0.5%
Enoxaparin Sodium; Sandoz	*M_w_* = 4350 ± 0 Da	M_2000_: 16.5 ± 1.0%
		M_2000–8000_: 81.0 ± 1.0%
		M_8000_: 2.5 ± 0.0%
Enoxaparin Sodium; Amphastar	*M_w_* = 4350 ± 27 Da	M_2000_: 13.5 ± 1.0%
		M_2000–8000_: 83.5 ± 1.0%
		M_8000_: 3.5 ± 1.0%

**Table 6:** Comparison of the technical aspects of each method.

	USP Method	Proposed Method
Required materials	USP Enoxaparin MW Calibrant A USP Enoxaparin MW Calibrant B USP Enoxaparin RS Enoxaparin sample	Oligosaccharide Standard Solution Enoxaparin sample
Required HPLC time ^1^	~5.3 h	~1.3 h
Analysis	Create calibration curve Determine system suitabiltiy: determine *M_w_* for each duplicate USP Enoxaparin RS Analyze sample: determine *M_w_* and distribution range for each duplicate enoxaparin sample	Input RT and peak area data from chromatogram into SVM model

^1^ Chromatograms should be recorded for a length of time sufficient for complete elution, including salt and solvent peaks. In this work, all samples were recorded for 40 min, and therefore the approximate total HPLC run time is calculated by multiplying the number of samples to be run (accounting for duplicate injections of each) by 40 min.

## Supplementary Materials

The following are available online at www.mdpi.com/1424-8247/10/3/66/s1. Figure S1：SEC chromatograms of synthetic oligosaccharides, Figure S2: (**A**) USP MW Calibrant A with provided MW values indicated; (**B**) USP MW Calibrant B with provided MW values indicated, Figure S3: Comparison of USP Enoxaparin Calibrants (red) and structural series (black) (**A**) NAc series (**B**) NS series (**C**) NS2S series (**D**) NS6S series (**E**) NS6S2S series, Figure S4: 310 nm trace from enoxaparin/oligosaccharide mixture (black) overlaid with 310 nm trace from oligosaccharide mixture alone (red). Chromatograms were obtained on different days and under the same HPLC conditions. Overlap of traces indicates that data from this system is suitable for use by the SVM model, Table S1: SVM data set parameters, Table S2: MW determination of largest components in USP Enoxaparin RS.
