# Supplementary material for: Modernization of Enoxaparin Molecular Weight Determination Using Homogeneous Standards
Source: Pharmaceuticals (Basel). 2017 Jul 22;10(3):66. doi: 10.3390/ph10030066 (PMC5620610; doi:10.3390/ph10030066)
Supplement: Supplementary file 1 [file pharmaceuticals-10-00066-s001.pdf]

pharmaceuticals

Supplementary Material: Modernization of Enoxaparin Molecular Weight Determination Using Homogeneous Standards

Katelyn M. Arnold, Stephen J. Capuzzi, Yongmei Xu, Eugene N. Muratov, Kevin Carrick, Anita Y. Szajek, Alexander Tropsha and Jian Liu

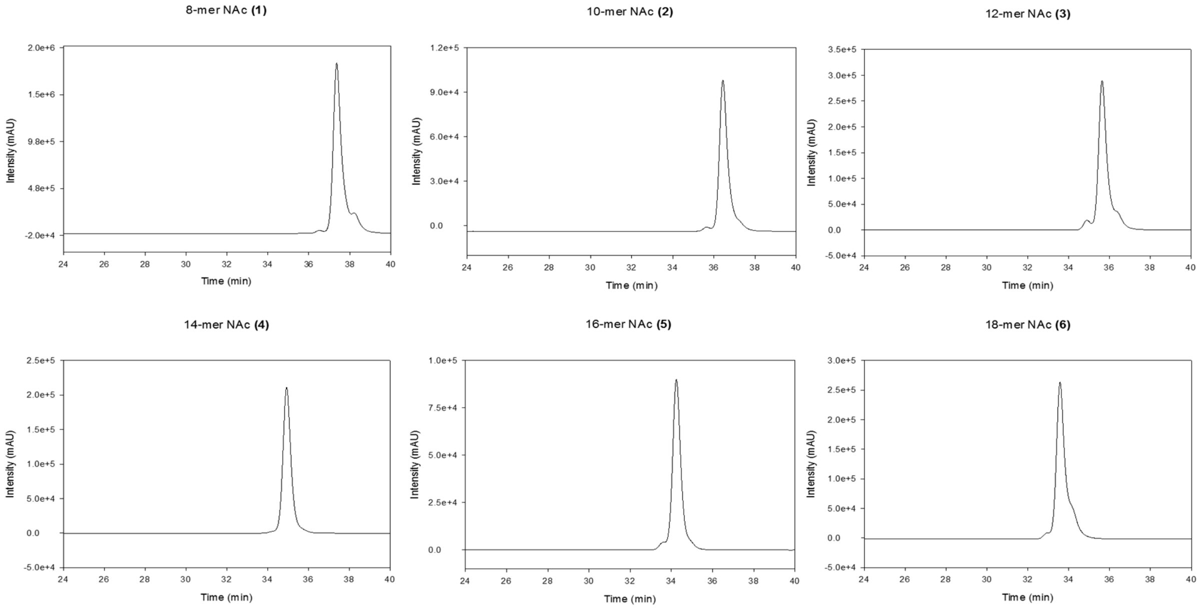

(A)

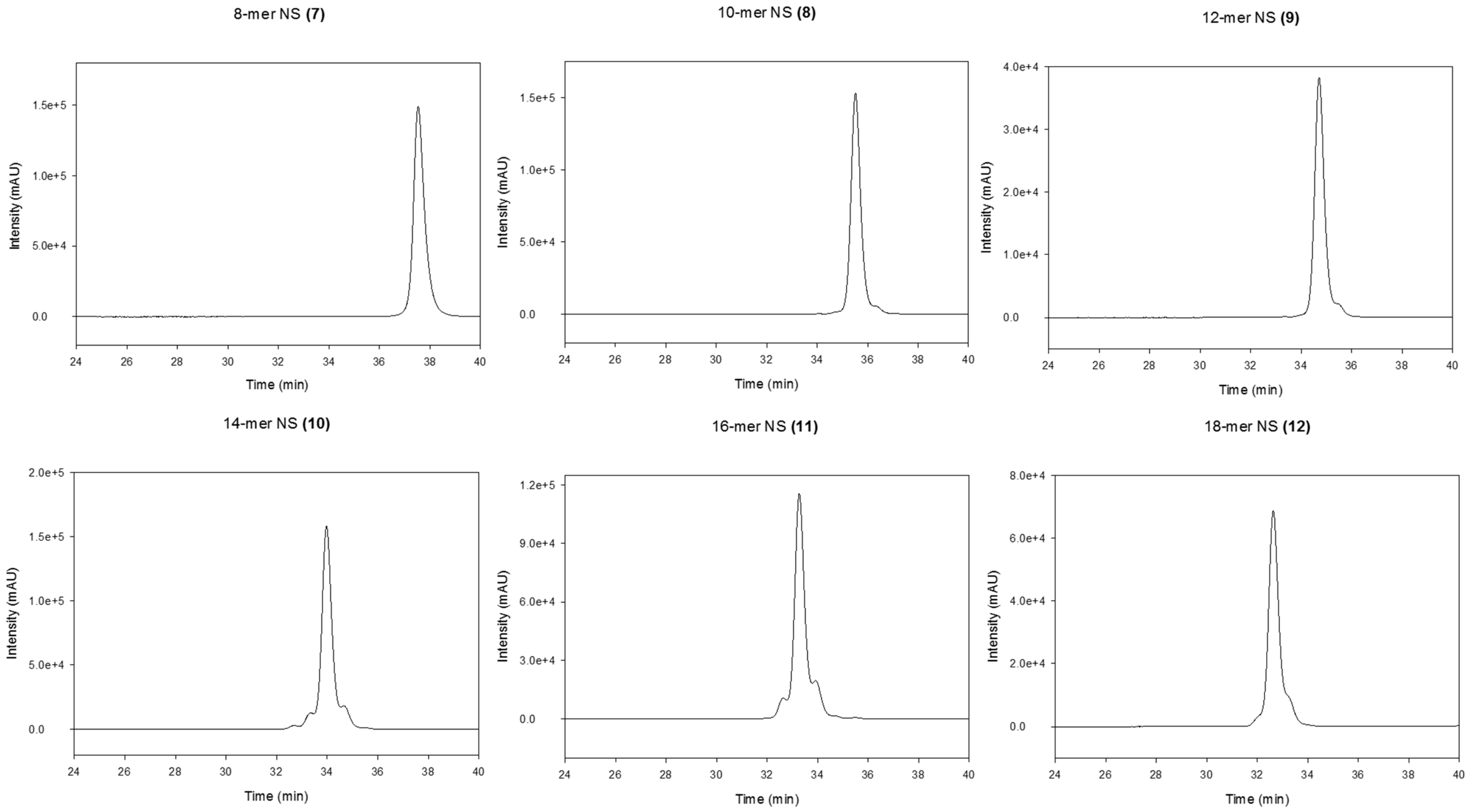
8-mer NS (7)
10-mer NS (8)
12-mer NS (9)
14-mer NS (10)
16-mer NS (11)
18-mer NS (12)
Intensity (mAU)
Time (min)

(**B**)

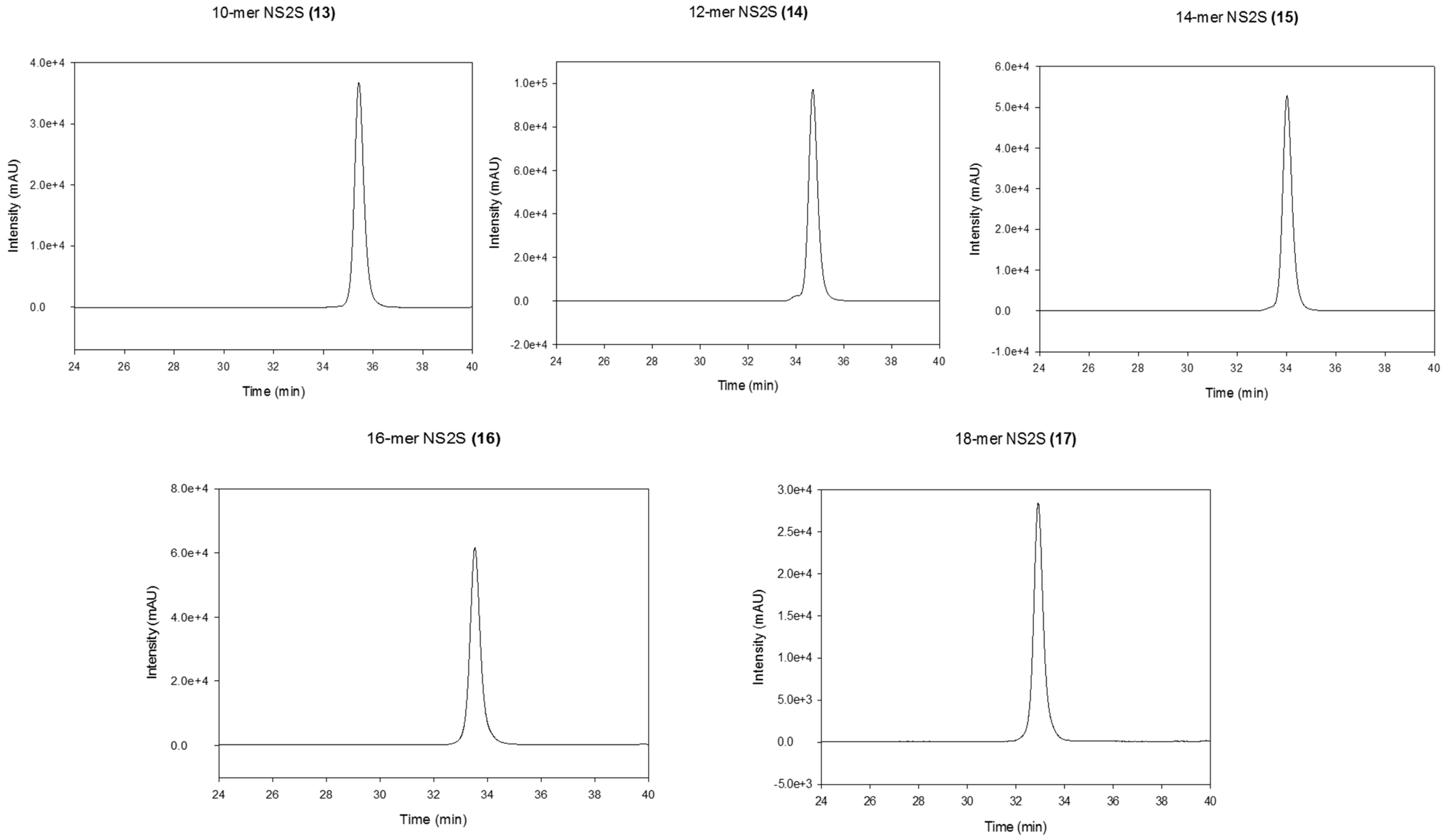
10-mer NS2S **(13)**
12-mer NS2S **(14)**
14-mer NS2S **(15)**
16-mer NS2S **(16)**
18-mer NS2S **(17)**
Intensity (mAU)
Time (min)

(**C**)

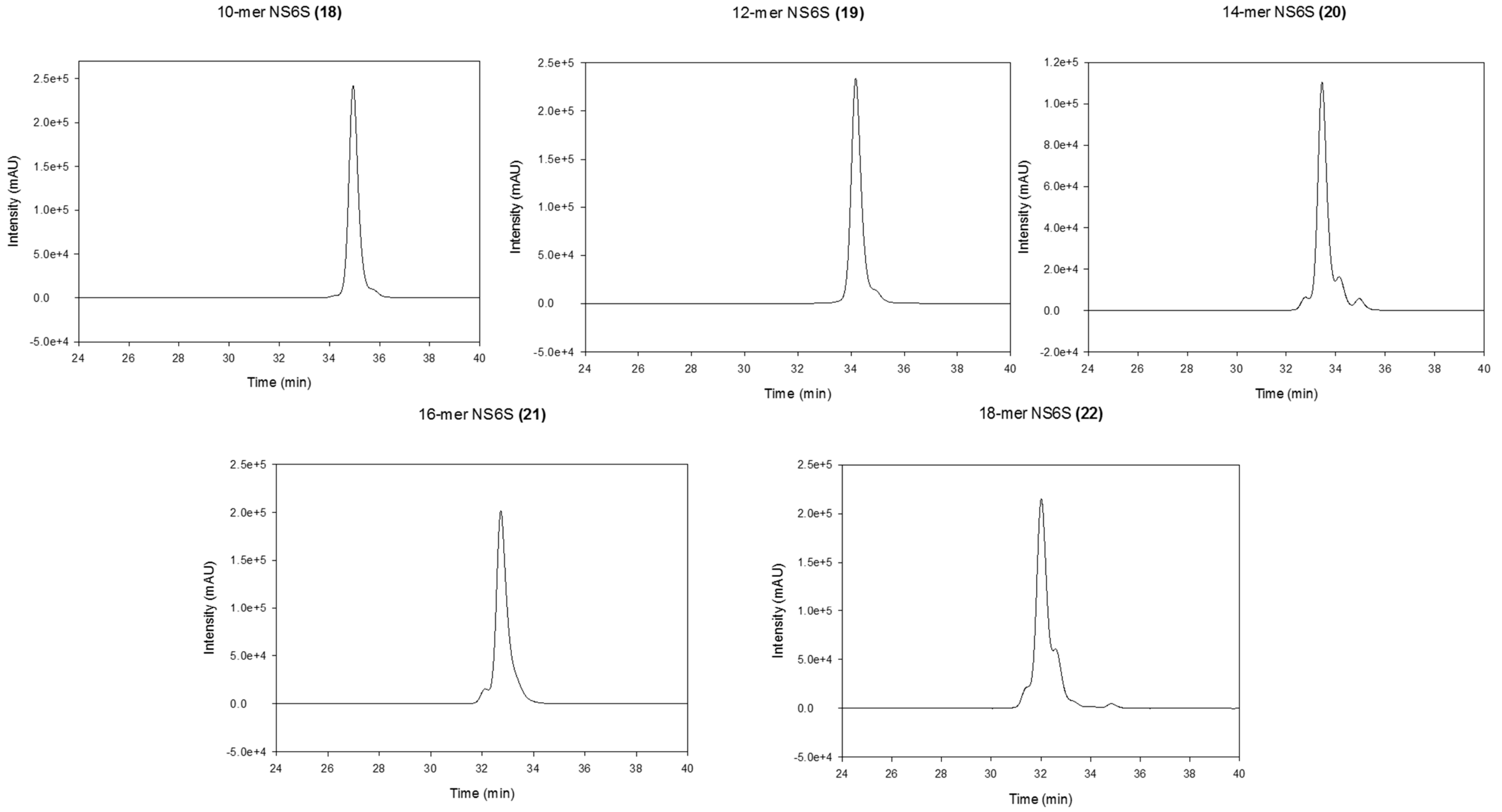
10-mer NS6S **(18)**
12-mer NS6S **(19)**
14-mer NS6S **(20)**
16-mer NS6S **(21)**
18-mer NS6S **(22)**
Intensity (mAU)
Time (min)

(**D**)

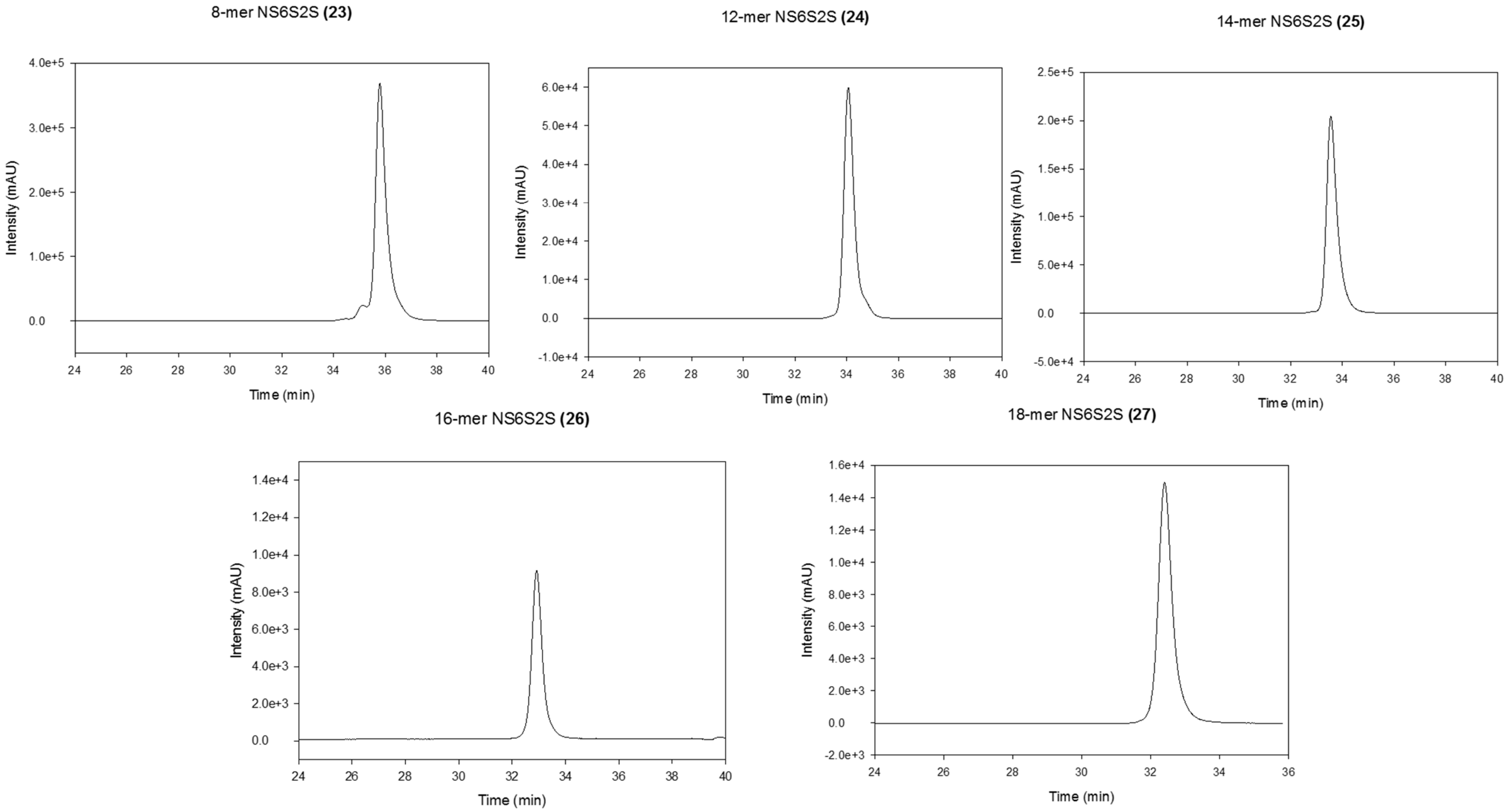

(**E**)

Figure S1. SEC chromatograms of synthetic oligosaccharides.

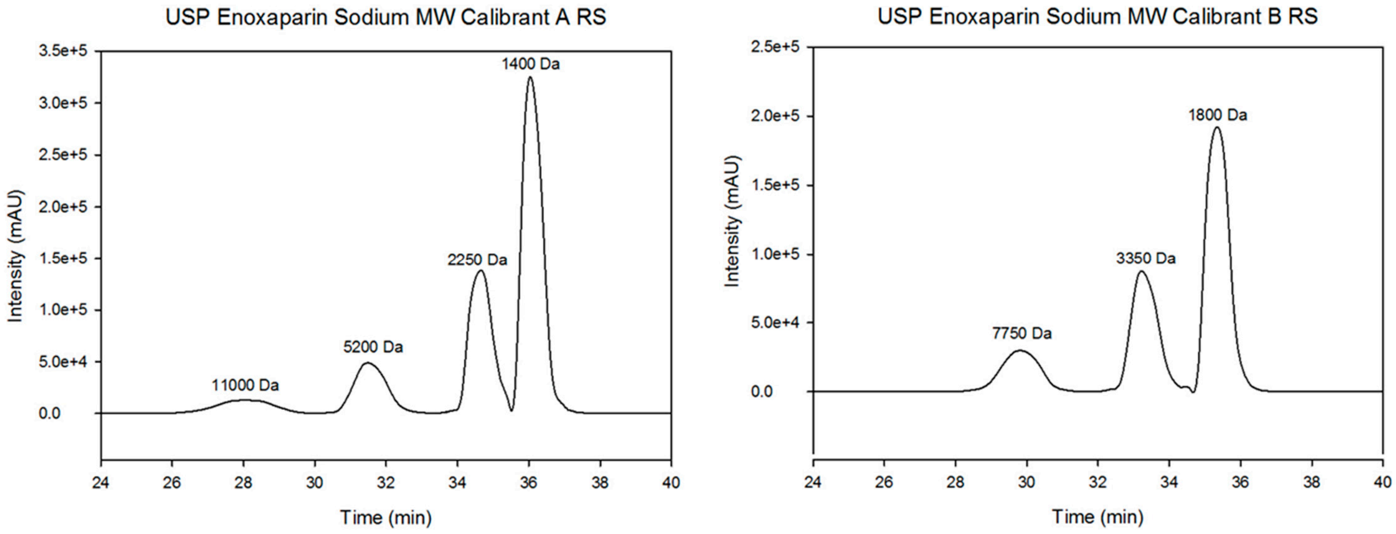

Figure S2. (**A**) USP MW Calibrant A with provided MW values indicated; (**B**) USP MW Calibrant B with provided MW values indicated.

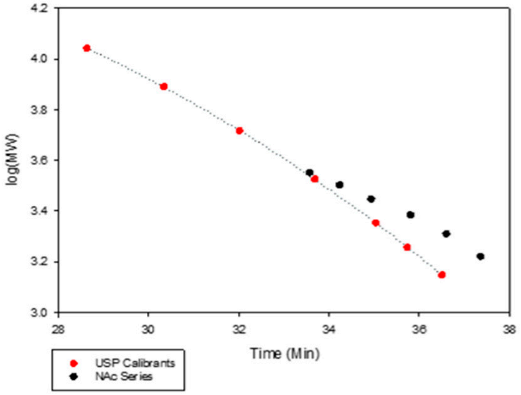

(**A**)

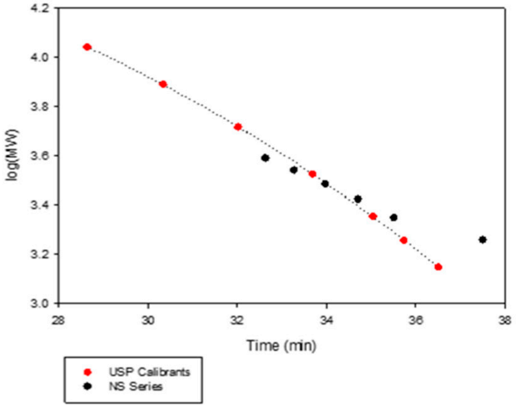

(**B**)

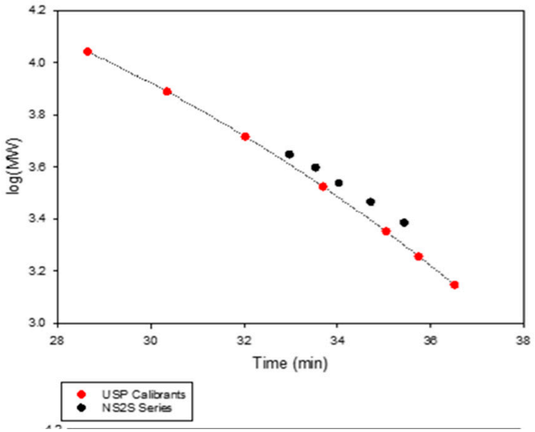

(**C**)

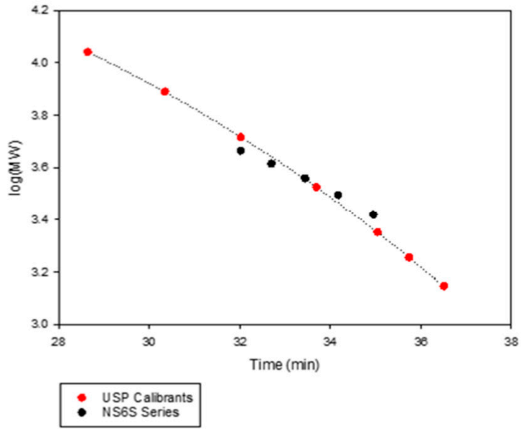

(**D**)

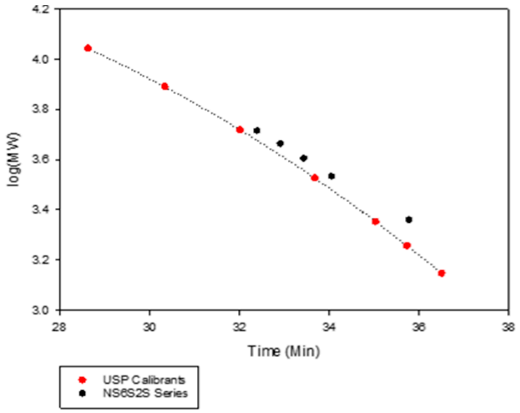

(**E**)

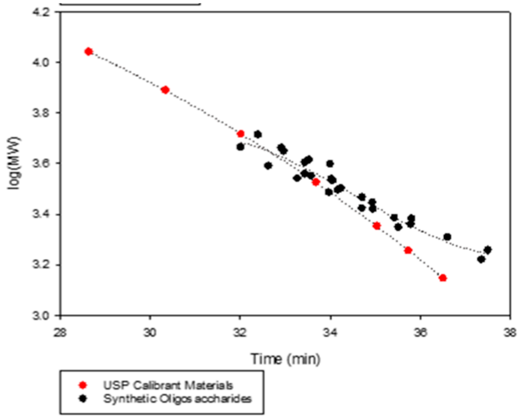

(**F**)

Figure S3. Comparison of USP Enoxaparin Calibrants (red) and structural series (black) (**A**) NAc series (**B**) NS series (**C**) NS2S series (**D**) NS6S series (**E**) NS6S2S series.

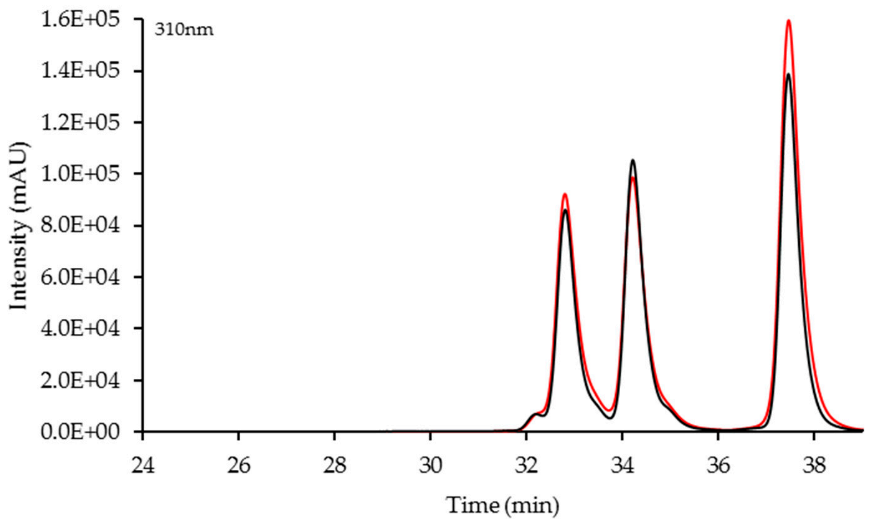

Figure S4. 310 nm trace from enoxaparin/oligosaccharide mixture (black) overlaid with 310 nm trace from oligosaccharide mixture alone (red). Chromatograms were obtained on different days and under the same HPLC conditions. Overlap of traces indicates that data from this system is suitable for use by the SVM model.

Table S1. SVM data set parameters.

Group	MW Range (Da)	RT Range (min)	Weight Factor
A (NAc series)	2415–3553	33.9–35.8	1
B (NS series)	2226–3895	32.6–35.5	1
C (NS2S series)	2466–4456	32.9–35.4	2
D (NS6S series)	2626–4616	32.0–34.9	4
E (NS6S2S series)	2289–5176	32.4–35.8	10
F (Dp4 & Dp6)	1200–1800	35.9–36.9	10
G (USP calibrants)	1400–11000	28.6–36.5	1

Table S2. MW determination of largest components in USP Enoxaparin RS.

Peak Number	RT (min)	Area %	MW Predicted Using USP Calibrants (Da)	MW Predicted Using SVM Model (Da)	Deviation of Average from USP Calibrants
1	26.8	0.038	16350 ± 1550	13550 ± 650	17% *
2	27.4	0.133	14850 ± 1400	12850 ± 700	13% *
3	27.9	0.271	13450 ± 1250	12050 ± 700	10% *
4	28.5	0.482	12050 ± 1050	11200 ± 700	6%
5	29.1	0.754	10800 ± 900	10300 ± 650	5%
6	29.6	1.130	9600 ± 750	9400 ± 550	2%
7	30.2	1.666	8500 ± 600	8500 ± 550	0%

* A deviation is seen with the largest peaks in Enoxaparin RS. Such difference is likely because different mathematical models were employed for the analysis.
